# Ancient evolutionary signals of protein-coding sequences allow the discovery of new genes in the *Drosophila melanogaster* genome

**DOI:** 10.1186/s12864-020-6632-y

**Published:** 2020-03-05

**Authors:** Carlos S. Casimiro-Soriguer, Alejandro Rubio, Juan Jimenez, Antonio J. Pérez-Pulido

**Affiliations:** Centro Andaluz de Biologia del Desarrollo (CABD, UPO-CSIC-JA). Facultad de Ciencias Experimentales (Área de Genética), Universidad Pablo de Olavide, 41013 Sevilla, Spain

**Keywords:** Ancient sequences, Gene finding, Protein-coding genes, *Drosophila melanogaster*, Protomotifs

## Abstract

**Background:**

The current growth in DNA sequencing techniques makes of genome annotation a crucial task in the genomic era. Traditional gene finders focus on protein-coding sequences, but they are far from being exhaustive. The number of this kind of genes continuously increases due to new experimental data and development of improved bioinformatics algorithms.

**Results:**

In this context, AnABlast represents a novel in silico strategy, based on the accumulation of short evolutionary signals identified by protein sequence alignments of low score. This strategy potentially highlights protein-coding regions in genomic sequences regardless of traditional homology or translation signatures. Here, we analyze the evolutionary information that the accumulation of these short signals encloses. Using the *Drosophila melanogaster* genome, we stablish optimal parameters for the accurate gene prediction with AnABlast and show that this new strategy significantly contributes to add genes, exons and pseudogenes regions, yet to be discovered in both already annotated and new genomes.

**Conclusions:**

AnABlast can be freely used to analyze genomic regions of whole genomes where it contributes to complete the previous annotation.

## Background

Research groups from all over the world are sequencing whole genomes as a common task, taking advantage of the current burst in the genomics era [1]. The analysis of the sequences from those genomes is essential for accurate annotation procedures. However, computational tools for gene discovery usually miss around 20% of protein-coding genes when annotating a whole genome, or even more in the case of eukaryotic organisms [2, 3]. Thus, a significant number of protein-coding sequences and other functional genomic elements are missing when using currently available genomic annotation approaches.

One of the most intensively studied model organism is the fruit-fly *Drosophila melanogaster*. Its genome was sequenced in 2000, and 13,601 protein-coding genes were initially annotated, coming from the integration of the two used gene finders, which respectively predicted 13,189 and 17,464 genes [4]. From this milestone, the number of fruit-fly genes has changed, and numerous and significant discrepancies have arisen [5]. But nowadays the FlyBase database put this number at 14,133 [6], showing that the number of genes is constantly increasing over time, and a greater increase is expected to come from the discovery of new kinds of genes, such as those shorter than 100 amino acids, which in the fruit-fly genome could account for thousands of them [7].

Traditional gene finders are routinely based on both significant sequence similarity and sequence signatures such as those used to define open reading frames (ORF), signals involved in splicing [8], or combined protocols to get better results [9]. Among the new proposed methods, we have previously shown that accumulation of low-score alignments, which would represent footprints of ancient sequences, highlights present and ancient protein-coding regions which are hard to discover by conventional methods [10]. Briefly, this novel computational approach, that we named AnABlast, compares the putative amino acid sequences from the six reading frames of a genomic sequence against a non-redundant protein database, and collects the matches, including low-score alignments, which we call protomotifs. These are specifically accumulated in coding but rarely in non-coding sequences. Thus, the profile of AnABlast with peaks of accumulated protomotifs, accurately marks putative protein-coding genes, pseudogenes, and fossils of ancient coding sequences, overcoming the effects of possible sequencing errors and reading frame shifts, since it does not search for reading frames but sequence coding signals.

We previously showed that this strategy is useful to discover putative protein-coding regions. It was tested with both intergenic and intronic sequences from the fission yeast *Schizosaccharomyces pombe*, and 18 putative genes were predicted [10]. But even though these predictions had computational support and some translation evidences, prediction parameters were empirically established by training the algorithm with S. pombe curated genes. Here, we use the whole well-studied *D. melanogaster* genome, and its annotated protein-coding regions to evaluate the method accuracy and determine optimal AnABlast parameters. The fine-tuning procedure was finally tested with an early version of a protein database, and we show that many new genes predicted by this algorithm are really true genes that have been incorporated into the current genome annotation of this organism. We also show that AnABlast is useful to discover small ORF and fossil sequences that are hidden to conventional gene finder algorithms, and show how this new strategy can contribute to discover the complete set of protein-coding regions of a whole genome.

## Results

### Searching for protein-coding signals in the fruit-fly genome

**Fig. 1:**
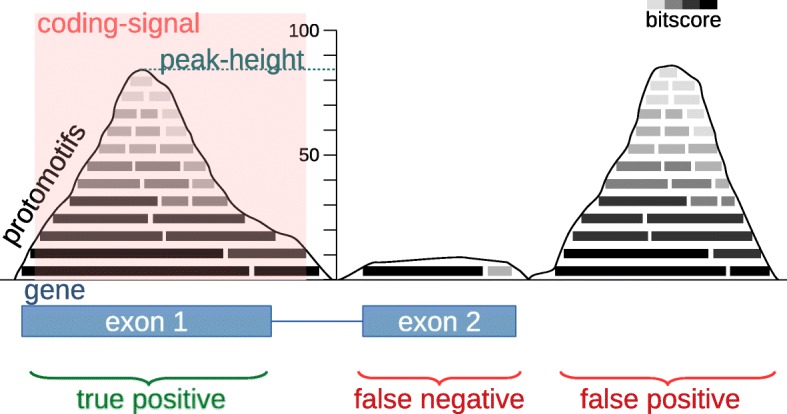
Schematic diagram showing AnABlast profiles obtained in a theoretical genomic region with a two-exon gene. The peak-height is the maximum accumulation of protomotifs in a specific genomic position (BLAST alignments including low bit-scores). Peaks with a protomotif accumulation above a peak-height threshold are considered as putative protein-coding regions (coding-signal). Significant peaks matching a known exon represents true positive peaks, while those underlying a genomic region without known exons are considered false positive coding-signals. Well-known exons which do not significantly accumulate protomotifs (peak-height below the threshold) constitute false negatives

AnABlast is a computational tool that searches for protein-coding regions in whole genomes by taking into account low-score alignments shared by multiple unrelated protein sequences. To this end, AnABlast uses the putative amino acid sequences translated from a genomic sequence to search for sequence similarity in a non-redundant protein database. Alignments obtained from this similarity search (called protomotifs), including those of a low score, are then piled up along the query sequence, and peaks accumulating protomotifs above a specific threshold will highlight potential protein-coding regions and will be considered coding-signals (Fig. 1). Finally, these coding-signals can be evaluated: those ones underlying exons from a protein-coding gene will be true positive predictions, exons without coding signals will be false negatives, and coding-signals underlying introns or intergenic regions will be putative false positives. But it should be noted that the false positives could potentially underlie new coding sequences which escaped to conventional annotation pipelines. Thus, false positives highlighted by AnABlast may represent genomic regions encoding putative new proteins, but also non-functional degenerated protein-coding regions, something of particular interest in current genome research.

To test the capability of AnABlast to discover protein-coding regions, we used this algorithm to analyze the whole genome of the fruit-fly *D. melanogaster* (2012 and 2017 releases). Protein sequences coming from the virtual translation of the complete fly genome (in all the six reading frames) were subjected to BLAST search against a non-redundant protein database (UniRef50) under a low restriction threshold, and the resulting alignments were accumulated along the query sequence to produce the AnABlast profile. Then, all well-known exons of this genome were compared with the set of putative coding regions identified by AnABlast. As expected, most of the AnABlast peaks with a high protomotif accumulation matched annotated exons (putative true positives), but a small fraction of them fell in both intronic and intergenic regions lacking of any annotated gene, exon or pseudogene (Suppl. file 1, genomic browser with AnABlast results). These false positive signals represent a particularly interesting set of genomic regions, since they could constitute new protein-coding regions.

### Protomotifs underlie into the true reading frame

**Fig. 2:**
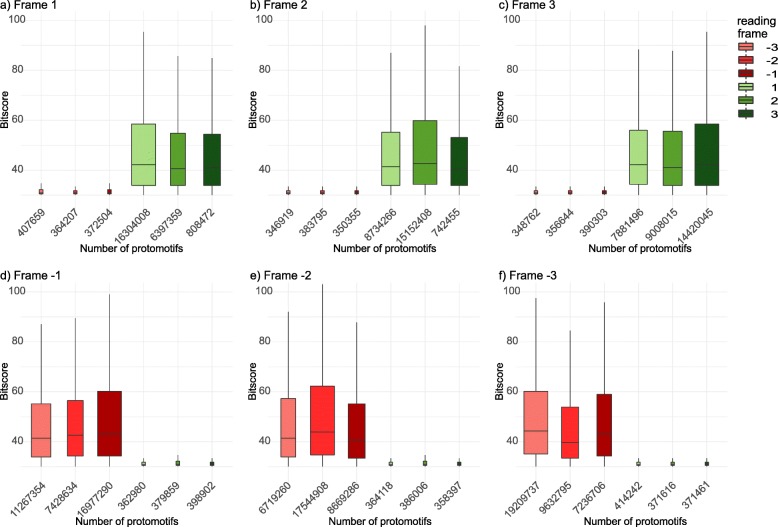
Distribution of protomotifs coming from true positive coding-signals separated by the true reading frame of the protein-coding sequences where they accumulate. The different parts of the figure represent protomotifs accumulated in protein-coding sequences at different BLAST bit-score starting in **a** frame + 1, **b** frame + 2, **c** frame + 3, **d** frame − 1, **e** frame − 2, and **f** frame − 3. The box size is proportional to the number of protomotifs in that frame, and the exact number of protomotifs is also shown below the X-axis. The three reading frames coming from the forward strand are colored in green color, and the three coming from the reverse strand are colored in red color

Protein-coding signals highlighted by AnABlast are mainly composed by protein sequence alignments of low score, but also occasionally high score. To test if such alignments are just random, or they actually match true protein-coding regions, we studied the distribution of protomotifs underlying protein-coding regions at different BLAST bit-scores, regarding to the different possible reading frames. Though millions of protomotifs were scattered throughout the fruit-fly genome within annotated exons, most of them were concentrated in the right reading frame, with a much lower number found in any of the other possible five reading frames (Fig. 2).

Thus, protomotifs are mainly accumulated in the true reading frame in spite of their low score. Interestingly, a significant number of them accumulate in the right strand but at a different reading frame. Contrarily, the other strand shows an enormously reduced number of protomotifs, with a lower order of magnitude. In fact, the protomotifs accumulated in the contrary strand present bit-score values lower than 30, but those accumulated in the true strand present values near to 100. This result suggests, from an evolutionary point of view, that new protein-coding genes might putatively come just from shifting the reading frame in the same strand.

### Optimization of AnABlast parameters for the efficient prediction of protein-coding signal

**Fig. 3:**
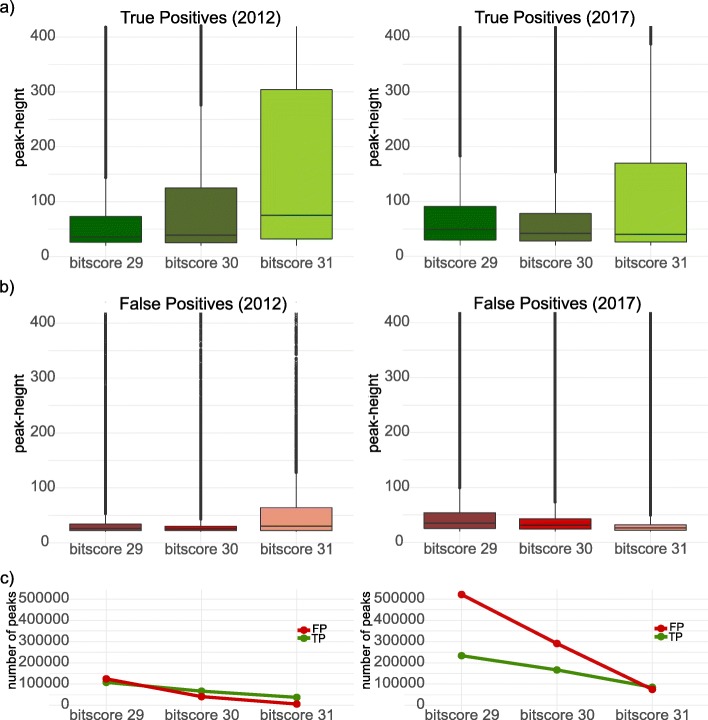
Peak-height distribution and number of coding-signals found at different bit-score values. Peak-height distributions are separated by **a** true positive and **b** false positive, and they are shown by each database release (2012 and 2017) and three different bit-score thresholds. The outliers are shown as a chain of points above the boxes. **c** The number of true and false positive coding-signals at any peak-height with the corresponding bit-score thresholds (note that it shows number of peaks with peak-heights as low as 20 and higher)

Until now we have seen how AnABlast coding-signals mainly match to protein-coding region in the genome, but we did not use any threshold to evaluate the results and measure the accuracy in the procedure of gene prediction. To optimize AnABlast parameters for the identification of new exons and genes, the distribution of both true and false positive coding-signals were evaluated at different peak-height thresholds. AnABlast profiles depend on the bit-score value used to restrict alignment significance during the BLAST search, therefore, in addition to the value of bit-score 30, previously used by AnABlast [10], the evaluation was carried out also using the more and less restrictive bit-scores of 29 and 31 respectively. Regardless of the taken score, true positive coding-signals account for the highest peak-height (Fig. 3), though under more restricted score values (higher bit-score), AnABlast peaks were more selective and focused into the protein-coding regions of the genome (higher peak-heights). However, the absolute number of true protein-coding regions dropped down with such higher scores, decreasing the number of peaks underling protein-coding sequences (Fig. 3c). On average, predicted coding-signals falling in non-coding regions (false positives) have much lower peak-height values (Fig. 3b). However, the distribution of these false positives shows outliers with peak-height values indistinguishable from the true positive set, which could be considered as new putative protein-coding regions.

In a specific genome, the accuracy of the protein-coding sequence prediction by AnABlast not only depends on the bit-score value, but also on the peak-height threshold coming from the alignment accumulation. Under a bit-score value of 30, the optimal peak-height cutoff depends on the used database and the amount of sequences that it contains. In this way, when the most current database from 2017 was used, the false positive outliers appear from a peak-height of 70, so proposing that peaks higher than this value could predict new protein-coding regions. However, when using the 2012 database, this peak-height value was around to 40.

**Fig. 4:**
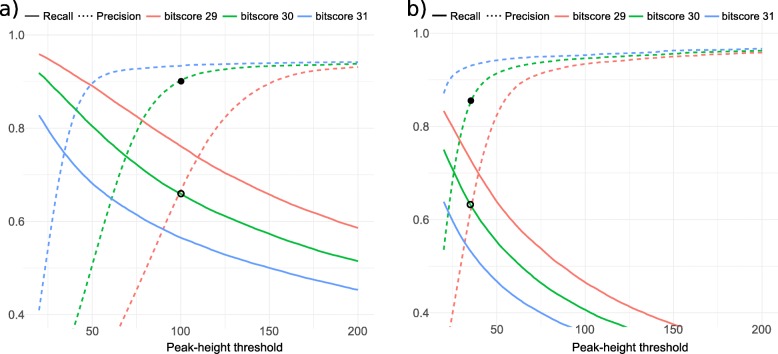
Recall and precision of AnABlast at different bit-score thresholds. Values were calculated when using the databases: **a** release 2017, and **b** release 2012. The black dot marks the precision value at bit-score 30, and the unfilled dot marks the recall. The complete results and values for all the used parameters can be found in Suppl. file 2

To better test both the precision and recall of AnABlast in predicting protein-coding sequences, coding-signals were compared against the gene annotation of fruit-fly genome and the accuracy of AnABlast prediction in this set was analyzed. As expected, the recall is higher when using a more recent database (release 2017, with more than 21 million proteins) compared to an older one (release 2012, with around 4.5 million proteins) at the same peak-height. When using the database of 2017, the precision has an asymptote at around peak-height equal to 100 with a value of around 90% (only 1 in 10 predictions are not right), though the recall at this threshold is only of 65% (only 6.5 in 10 of the true protein-coding sequences are recovered) (Fig. 4a). However, this accuracy is reached with peak-height equal to 35, when the older release of the database is used (Fig. 4b).

As described above, the precision varies regarding to the bit-score threshold used, and a higher precision and lower recall are reached when more restrictive values are used. So, to ensure a high accuracy we chose bit-score 30 and a peak-height threshold of 100. By using these parameters, we expect that AnABlast could discover new unknown protein-coding genes and exons inside the 10% putative false positives. However, with the older database, with a number of sequences almost five times lower, we should take a peak-height threshold of 35. All of this gives a great number of AnABlast coding-signals matching with protein-coding region spread over the fruit-fly chromosomes, and between 4500 and 7000 (depending of the used database) candidates to be new protein-coding sequences (Table 1; Suppl. file 3). These restricted parameters allow finding more than 30,000 exons from the current fruit-fly annotation.

**Table 1:** Number of true and false positives predicted by AnABlast using the 2012 and 2017 databases, and separated by chromosomes. The peak-height threshold used was 40 (2012) and 100 (2017)

	Chromosomes							
Coding-signals	2 L	2R	3 L	3R	4	X	Y	Total
True positives (2012)	6691	7515	6954	9085	387	6211	81	36,924
False positives (2012)	1035	1390	1384	1517	40	1143	483	6992
True positives (2017)	6139	6798	6164	8325	379	5390	72	33,267
False positives (2017)	535	1068	947	913	41	443	518	4465

### AnaBlast is able to discover current genes using an old database

The number of annotated protein-coding sequences is continuously revisited in annotated genomes, and new genes, exons and pseudogenes continuously appear as a consequence of experimental results and new in silico approaches. For instance, when the FlyBase database released in 2012 is compared to the current 2017 release, it can be found that 38 protein-coding genes, 91 exons from well-known genes, and 74 pseudogenes entered into the database later than 2012. This dataset of true protein-coding sequences absent in the 2012 allows us to carry out a simulation to estimate the efficiency of AnABlast in discovering new protein-coding sequences. Remarkably, when using the 2012 FlyBase database, with the parameters previously suggested (bit-score 30 and peak-height 35), we found that AnABlast highlights the majority of the protein-coding sequences from this dataset (Table 2). More than 60% of the protein-coding genes are found, and also the 80% of the pseudogenes were predicted by AnABlast. These results improve when using a less restricted peak-height value. In the case of new exons, their small length (some of them are coding for only a few amino acids) makes extremely difficult the in silico identification. However, up to 11% of them were also discovered by AnABlast, increasing up to 60% when changing the default peak-height to 26, which present a precision of 70% (Table 2; Suppl. file 4). Overall, it is important to highlight that the most of these new protein-coding sequences predicted by AnABlast were not found by the widely used gene finder AUGUSTUS [11].

**Table 2:** Genomic elements from the database release 2017 discovered using the database release 2012, separated by peak-height threshold. Note that ‘< 5’show the false negatives (when less than 5 protomotifs were found), and the last column show the most significant true positives

Sequence type	Annotated (2017)	Found by AUGUSTUS gene finder	Peak-height (< 5)	Peak-height (< 26)	Peak-height (26–35)	Peak-height (> 35)
Protein-coding gene	38	9	0 (0%)	14 (37%)	1 (2%)	23 (61%)
Exon	91	9	12 (13%)	55 (60%)	14 (16%)	10 (11%)
Pseudogene	74	15	0 (0%)	7 (9%)	8 (11%)	59 (80%)

**Fig. 5:**
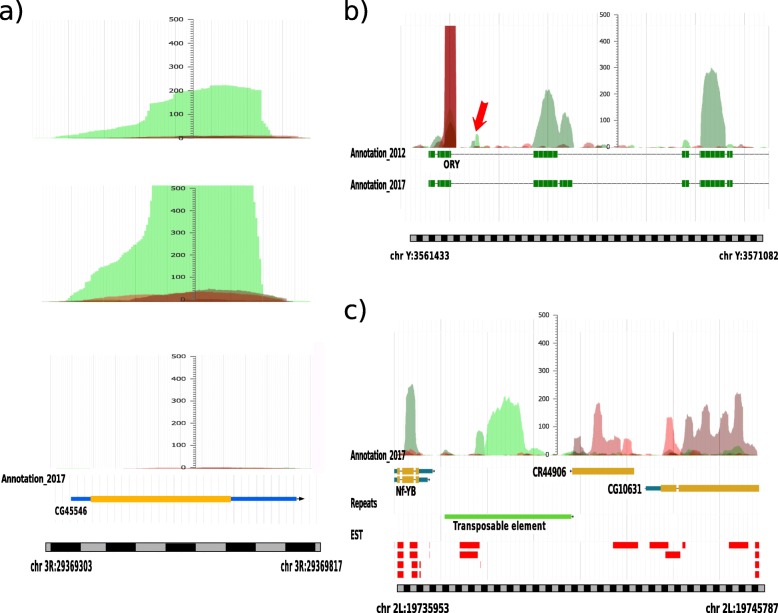
AnABlast profile for three regions of the fruit-fly genome. Green color represents protomotif accumulation in the forward strand, and red color in the reverse strand. **a** Different analysis for the gene CG45546 region, from top to bottom: using database release 2012, 2017 and using the reverse sequence as random query; **b** region including part of the gene *Ory* (CG40446), together with the exons annotated in both database releases (the red arrow marks two peaks corresponding to an ancient mobile element); **c** region of the pseudogene CR44906, including surrounding genes and a transposable element in the 5′ end. An additional track with EST signals (Expressed Sequence Tags) is shown, which suggests expression for the transposable sequence

The identification of very small genes is still challenging for in silico strategies, including AnABlast. One of the new genes that AnABlast failed to identify in the 2012 database (CG45546) is coding for a short protein of 93 amino acids (Fig. 5a). Interestingly, AnABlast efficiently identified it when using the 2017 database, due to the fact that this sequence and its putative homologs were now included in the database, increasing the peak-height to a significant level. This gene is still lost by AUGUSTUS, even when using the current database release. To discard that these coding-signals underlined by AnABlast occur by chance, the reverse sequence of this gene was used as a negative control. When this control is analyzed, AnABlast profiles present no accumulation of protomotifs (Fig. 5a, below). Furthermore, we shuffled the sequence of the gene, and the 85% of the simulations did not present any protomotif, and the remaining 15% gave peak-height values lower than 18 (Suppl. file 5).

Some new exons are also found and highlighted within well-annotated genes. One of these exons was found in the *Ory* gene (CG40446). The exon appearing in the 2017 database is found by AnABlast using the 2012 release and a peak-height higher than 35 (Fig. 5b). Interestingly, AnABlast produced two weak peaks within an intronic sequence of this gene, with a peak-height around 50, very similar to others in the 3′ end. Conventional search for homologous sequences to these AnABlast coding-signals revealed similarity with retrotransposons from invertebrate organisms, suggesting that this genomic region is coding for ancient proteins of a mobile element. In addition, a high peak overlapping with an exon in the 5′ end is also emerging in the reverse strand, which represents a tri-nucleotide region coding for amino acid repeats in all the reading frames. This artefact is characteristic of nucleotide repeats, and it can be avoided by enabling the low-complexity filter in the similarity search step with BLAST.

In addition to new genes and exons, AnABlast was able to discover 59 pseudogenes which did not appear in the used 2012 database (Table 2). This shows the ability of AnABlast for discovering protein-coding regions regardless of the presence of a complete open reading frame. One of these pseudogenes (CR44906), included in FlyBase in 2013, is clearly highlighted by AnABlast in the reverse strand of the 2012 database (Fig. 5c). Remarkably, another coding-signal is found in the forward strand, upstream of this pseudogene. In deep analysis of this sequence revealed that it encodes the transposase of an annotated transposable element. The presence of numerous expression sequence tags (EST) support the expression of this sequence. However, this transposase is not yet annotated in FlyBase database.

### Putative new protein-coding sequences in the present database

**Fig. 6:**
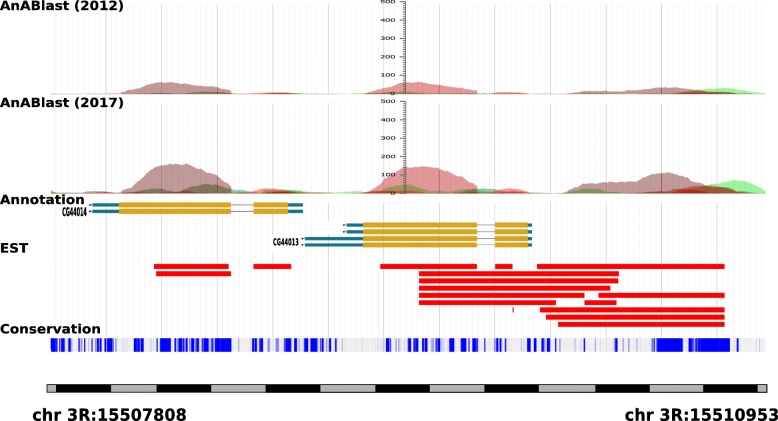
AnABlast profile for a region with a putative new gene. The profiles were created with both 2012 and 2017 release databases, and they are shown together with the gene, the EST track and one additional track taken from the UCSC browser representing the evolutionary conservation of the sequence versus 27 different insect genomes, which shows a high conservation for the proposed new gene

Finally, according to the efficient identification of protein-coding sequences highlighted by AnABlast, it is expected that after a future further characterization, a considerable fraction of the false positive sequences predicted when the 2017 database is used become true positives. One of these candidates is found 3′ upstream to the genes CG44014 and CG44013, coding for uncharacterized proteins bearing a calycin domain related to extracellular proteins and involved on lipid transport. AnABlast suggests a significant coding-signal in this region (Fig. 6). The putative protein sequence encoded by this AnABlast region has no homologues in other organisms, but it is located in an evolutionary conserved region, again matching with EST signals which also support the putative expression of this genomic region. A list of false positives which could likely propose new putative protein-coding sequences is available in Suppl. file 3, and in tracks FP (False Positives) in Suppl. file 1.

## Discussion

Currently, gene finder algorithms have a limited recall and usually lose the 10–20% of the true coding regions [3], especially those lacking homologs and/or having non-conventional characteristics such as small ORFs or pseudogenes. It leads to the necessary development of new algorithms based on different ideas [12, 13]. In this context, we proposed a new in silico strategy, named AnABlast that uses low-score alignments coming from multiple non-redundant proteins [10]. As shown in this study, in agreement with previous reports [14–16], these alignments (protomotifs) do not accumulate randomly but in true genomic protein-coding regions (Supplementary file 1; Fig. 3). So, it is expected that future growth in the protein database will increase the protomotif accumulation in true protein-coding regions, but none accumulation would appear in non-coding regions.

By using the *D. melanogaster* genome as a model system, we set optimal parameters for using AnABlast as a new protein-coding finder in whole sequenced genomes. But, AnABlast has not the aim of annotating an entire genome, since it allows the identification of only 60–85% of the actual genes annotated in a genome (Table 2). However, the accumulation of protein sequences using a non-redundant database and low bit-scores in the BLAST search enables AnABlast to discover new genes that scape to conventional strategies, producing a precision up to 90% with exons, genes and pseudogenes among the identified protein-coding signals (Fig. 4). Thus, AnABlast is particularly useful to re-search for new genes in already annotated genomes. Another advantage of the AnABlast strategy is the fact that protomotifs are accumulated within the true reading frame, and not scattered throughout the genome (Fig. 2). Importantly, coding signals are also identified by AnABlast in the coding strand, but at different reading frames. It rarely occurs in any of the three reading frames coming from the reverse strand, suggesting that new protein-coding exons or genes may emerge by frameshift mutations in preexisting ORFs [17]. In fact, the peak-height distribution matching ORFs in true genes has the highest value, followed by peaks identified in the next frame, which suggests that new protein-coding regions may emerge by point deletions in the original frame. This observation agree with previous evidences in mammals suggesting a higher frequency of evolutionary fixation for deletion than for insertion mutations [18, 19], a trend that have also been found in the *D. melanogaster* genome [20]. Remarkably, AnABlast coding-signal are sometimes found in the ends of well-known genes, overlapping with the right reading frame and suggesting than C-terminus and N-terminus of genes are subjected to evolutionary contractions and expansions that are efficiently identified by AnABlast [10].

The discovery of protein-coding genes by AnABlast is independent of the appearance of an open reading frame, a feature that allows finding sequences without canonical structures, such as pseudogenes and transposable sequences. Disabled or unitary pseudogenes originated from inactivated genes are particularly difficult to identify due to its high sequence divergence after long-term evolution [21]. Since AnABlast searches for the accumulation of footprints of common ancient protein sequences (low-score patterns), this strategy is particularly useful in underlying fossil sequences in which significant homology is lost (Fig. 5c).

Another important challenge to the whole annotation of genomes is the discovery of short ORFs [7]. These short protein-coding sequences were missed in the past, since it is difficult to distinguish between functional open reading frames and non-functional ones arisen by chance [22]. Albeit less efficiently, AnABlast is also useful for assisting in this task (Fig. 5a). Altogether, we encourage the use of AnABlast as a good in silico method that complement current gene finder algorithms and conventional genome annotation tools.

## Conclusions

The present study shows how AnABlast is able to discover new putative protein-coding genes and exons where other methods fail. AnABlast is also able to locate pseudogenes showing evolutionary remnants or even small ORFs that escape the conventional searches. Predictions were validated with a strategy that uses a recent database version, independent of the database used to carry out the gene finding. This could constitute a useful protocol to test computational gene finders.

All these features make of AnABlast a meaningful tool for the exhaustive analysis of genomic data, currently produced at an increasingly rapid rate. To allow the analysis of genomic regions, we have built a web application which is available at http://www.bioinfocabd.upo.es/anablast/ [23], where researchers can evaluate new protein-coding signals in their own sequences. Our results aim to analyze new genomes as well as to revisit annotated ones in order to discover new hidden genes.

## Methods

### Search for protein-coding signals

AnaBlast was used to search for protomotifs using the release 6 from *D. melanogaster* genome versus the UniRef50 database from January 2012 (with 4,606,913 sequences) and January 2017 (with 21,859.863 sequences), independently. UniRef50 is a protein database with non-redundant sequences in 50% identity threshold [24]. Blastx was used to get hits (that we call protomotif) with a threshold e-value of 10 and a bit-score between 29 and 31, which gave significant results in other projects [10], though the e-value has been decreased in order to optimize the analysis of a complete genome.

Protomotifs were classified by reading frame, when they matched to well-known exons in the genome. The distribution of protomotifs was made using the ggplot library of R programming language.

The genome analysis was performed in an HPC cluster, using 100 threads and it lasted around 1 week. The remaining analysis with sequences up to 10 kb were performed in the web application of AnABlast, which allows to analyze genomic sequences up to 25Kb, or longer if the user provides the precalculated BLAST report: http://www.bioinfocabd.upo.es/anablast/.

### Testing protocol

The *D. melanogaster* genome annotation release dmel-all-r5.43 from January 2012 was converted to the release dmel-all-r6.19 from January 2017 using the conversion tool from the FlyBase database. Genes were compared with bedtools intersect, obtaining all the new exons, complete genes, and pseudogenes appearing in release 6 but not in release 5. To a higher constraint, the sequences were searched in UniProt database to discard previously described protein-coding genes, and only genes not appearing in any database release before 2017 were maintained. The remaining sequences were taken and used as the testing dataset. For the testing protocol, Blastx was run with the genome release 6 and the Uniref50 database release from January 2012. The sequences from the testing dataset were taken with 100 nucleotides both in the 5′ and 3′ ends, previous to analyze by AnABlast.

Both tracks for EST sequences and conservation (27 insects conservation by PhastCons) were obtained from the UCSC browser [25].

### Accuracy measurement

A coding-signal is considered to match with an annotated exon when at least the 20% of the exon is covered, or the 20% of the peak underlies the exon. To check this, AnaBlast results were converted to bed format and compared to the GFF file with the annotated genes from the *D. melanogaster* release 6. Accuracy was measured by comparing exons and protein-coding signal from AnABlast, considering true positives (TP, AnABlast coding-signals matching to exons, or pseudogenes), false positives (FP, AnABlast coding-signals matching to introns or intergenic regions), and false negatives (FN, exons or pseudogenes without AnABlast coding-signals). Then, precision (specificity of the analysis: percentage of right predictions in the results) and recall (sensitivity of the analysis: percentage of right elements which are predicted) was calculated:

$$ \mathrm{Precision}=\left(\mathrm{TP}/\left(\mathrm{TP}+\mathrm{FP}\right)\right)\times 100 $$

$$ \mathrm{Recall}=\left(\mathrm{TP}/\left(\mathrm{TP}+\mathrm{FN}\right)\right)\times 100 $$

## Supplementary information

**Additional file 1.** Genomic browser which shows all the obtained results compared with the reference annotation from FlyBase database. It includes the following tracks: FlyBase genes, AnABlast profiles using 2012 and 2017 database, EST sequences from UCSC, and both true and false positives with a peak-height threshold of 35 (2012) and 100 (2017). http://www.bioinfocabd.upo.es/drome/.

**Additional file 2.** Recall and precision values when using AnABlast at different peak-height thresholds with the fruit-fly genome and the 2012 and 2017 releases of the database.

**Additional file 3.** List of all true and false positives predicted by AnABlast using both 2012 and 2017 databases. The peak-height threshold used was 35 (2012) and 100 (2017). Note that false positives from 2017 are novel predicted protein-coding sequences.

**Additional file 4.** AnABlast results for all the sequences in the testing dataset, including genes, exons and pseudogenes. It is shown if the genomic element is found by the gene finder AUGUSTUS.

**Additional file 5.** AnABlast results for 100 shuffled versions of the CG45546 gene, in addition to its reverse sequence.

## Data Availability

All data generated or analyzed during this study are included in this published article and its supplementary information files.

## Abbreviations

FN: False negatives
FP: False positives
ORF: Open reading frame
TP: True positives
